# Th1/M1 Conversion to Th2/M2 Responses in Models of Inflammation Lacking Cell Death Stimulates Maturation of Monocyte Precursors to Fibroblasts

**DOI:** 10.3389/fimmu.2013.00287

**Published:** 2013-09-18

**Authors:** JoAnn Trial, Katarzyna A. Cieslik, Sandra B. Haudek, Clemens Duerrschmid, Mark L. Entman

**Affiliations:** ^1^Division of Cardiovascular Sciences, Department of Medicine, DeBakey Heart Center, Baylor College of Medicine, Houston, TX, USA; ^2^Houston Methodist, Houston, TX, USA

**Keywords:** IL-13, monocytes, fibrosis, IL-33, MCP-1, M1, M2

## Abstract

We have demonstrated that cardiac fibrosis arises from the differentiation of monocyte-derived fibroblasts. We present here evidence that this process requires sequential Th1 and Th2 induction promoting analogous M1 (classically activated) and M2 (alternatively activated) macrophage polarity. Our models are: (1) mice subjected to daily repetitive ischemia and reperfusion (I/R) without infarction and (2) the *in vitro* transmigration of human mononuclear leukocytes through human cardiac microvascular endothelium. In the mouse heart, leukocytes entered after I/R in response to monocyte chemoattractant protein-1 (MCP-1), which is the major cytokine induced by this protocol. Monocytes within the heart then differentiated into fibroblasts making collagen while bearing the markers of M2 macrophages. T cells were seen in these hearts as well as in the human heart with cardiomyopathy. In the *in vitro* model, transmigration of the leukocytes was likewise induced by MCP-1 and some monocytes matured into fibroblasts bearing M2 markers. In this model, the MCP-1 stimulus induced a transient Th1 and M1 response that developed into a predominantly Th2 and M2 response. An increase in the Th2 product IL-13 was present in both the human and the mouse models, consistent with its known role in fibrosis. In these simplified models, in which there is no cell death to stimulate an anti-inflammatory response, there is nonetheless a resolution of inflammation enabling a profibrotic environment. This induces the maturation of monocyte precursors into fibroblasts.

## Introduction

The immune system uses similar tools whether responding to infections or sterile tissue injury. The same steps of an inflammatory response followed by resolution and healing take place under both circumstances (1). For example, the liberation of heat shock proteins (2), mitochondrial DNA (3), HMGB1 (high-mobility group box 1) (4, 5), and a host of other internal components from dead cells [alarmins (6)] can trigger inflammation in the absence of infection. Others, such as adenosine (7), apoptotic cell membranes (8, 9), or self DNA (10), can at the same time initiate the anti-inflammatory response that will bring the organism back to homeostatic balance. Both types of influences may act via chemokines that attract different subsets of immunocytes or may skew the subsequent maturation of the cells (11). In contrast to that schema, this report describes and defines similar responses, both proinflammatory and anti-inflammatory, in the absence of cell death and its associated signaling.

Our murine model of intermittent cardiac ischemia and reperfusion (I/R) *in vivo*, despite the absence of cell death, still results in an immune response. This treatment, which leads to cardiomyopathy, is the daily repetitive brief (15 min) I/R of a coronary artery, in which the major chemokine induced is monocyte chemoattractant protein-1 (MCP-1) and histology reveals interstitial fibrosis but no necrosis (12). We have shown that the fibrosis results from an influx of CD34^+^CD45^+^ monocytes that become fibroblasts making collagen (13). As in many models of fibrosis, these cells bear the markers of M2 macrophages while also assuming the shape and markers of fibroblasts (14). Because this model does not result in the death of cardiomyocytes, the mediators inducing the monocyte influx and transition into fibroblasts are limited in comparison to those accompanying a myocardial infarct, including both proinflammatory and anti-inflammatory or profibrotic influences (12). Neither the influx nor the fibrosis occurs in animals when MCP-1 is genetically deleted, indicating the necessity of this chemokine for initiation of the immune response leading to fibrosis (15, 16).

To simulate these events using human cells *in vitro*, we designed a transendothelial migration (TEM) assay using human cardiac microvascular endothelial cells (HCMEC) growing on a model basement membrane over an insert with 8 μm pores. Human mononuclear cells are attracted to migrate through the endothelium and the pores in response to MCP-1 below the insert (17). Some of the transmigrating monocytes become M1 macrophages, whereas others become M2 macrophages that mature into fibroblasts, and the numbers of the latter vary with different stimuli. We have used this TEM model to investigate the response of monocytes to Fc receptor engagement (17), angiotensin-II (18), and ROCK-1 knockdown (19), with parallel results to the corresponding *in vivo* mouse models. As with the *in vivo* models, MCP-1 is necessary for the migration of the monocytes through the endothelium (17). Classically, MCP-1 is regarded as a stimulus for M1 development, but MCP-1 may be accompanied by other chemokines to which a subset of monocytes capable of maturation into M2 macrophages may migrate (20). A major inducer of the M2 phenotype is the Th2 cell product, IL-13 (11), and addition or production of IL-13 is required for the formation of monocyte-derived fibroblasts in our *in vitro* assay (21). This suggests that it may be pertinent to human disease states.

Since IL-13 has been associated with fibrosis in the aging heart and in other organs (21–23), we elected to investigate its presence and kinetics in our cardiomyopathy model as well as in our *in vitro* model. Although IL-13 can be made by many kinds of cells, the source of it in an inflammatory infiltrate is likely to be predominantly from T cells, and so we verified the presence of T lymphocytes. Our data suggest that the presence of a Th2 response is integral to the fibrosis in these models of immune activation, in which there is an initial induction of the Th1 and M1 responses that then evolve into a Th2/M2 response in the absence of cell death. The Th2 response is obligate for the formation of myeloid fibroblasts and the development of a fibrotic cardiomyopathy.

## Results

### Murine *in vivo* model

**Figure 1:**
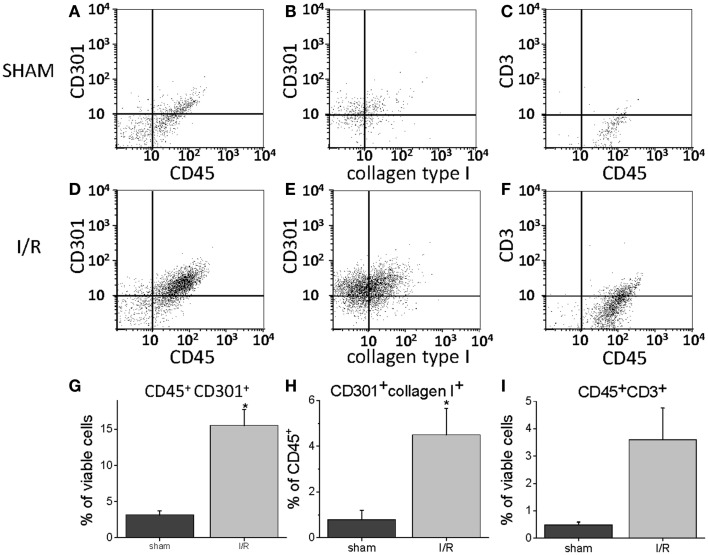
**Flow cytometry for the indicated markers on cells harvested from mouse hearts after 5 days of I/R or from sham-treated animals**. **(A)** Through **(C)** are representative histograms for sham-treated animals. **(D)** Through **(F)** are representative histograms for I/R animals. **(G)** Through **(I)** are summary data graphs. **(A,D,G)** Represent the percent of all viable cells (calcein positive) that were positive for both CD45 and CD301; **(B,E,H)** are the percent of all cells positive for CD45 that were also positive for both CD301 and collagen type I; **(C,F,I)** are the percent of all viable cells that were positive for both CD45 and CD3 (*n* = 3, *p < 0.05).

**Figure 2:**
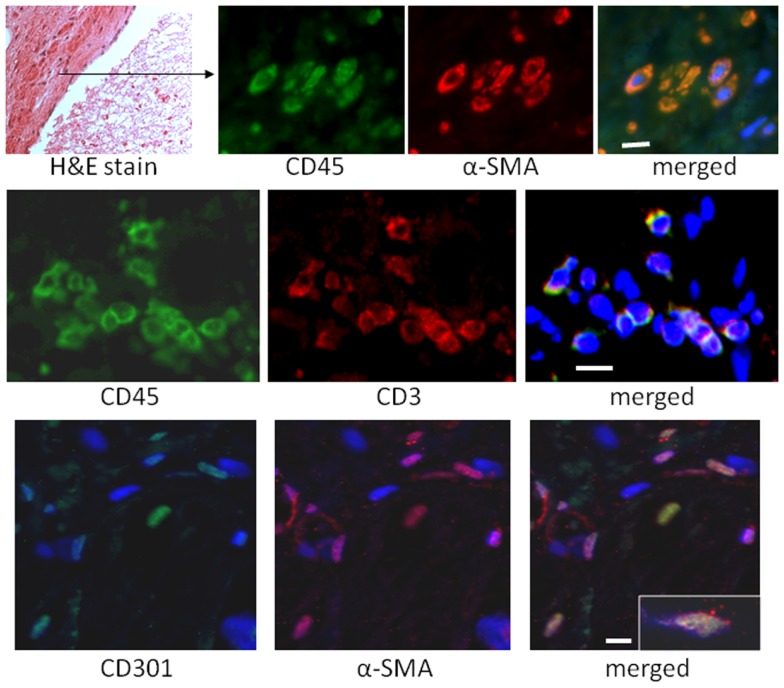
**Sections of human hearts with cardiomyopathy were stained with hematoxylin and eosin (H&E) or by immunofluorescence for the indicated markers**. The white bars are 10 μm.

To quantify the number of M2 macrophages and T lymphocytes in heart tissue from shams or animals treated with the I/R protocol, we digested the heart to isolate cells and performed flow cytometry to measure the expression of the markers CD45 (cells of hematopoietic origin), CD301 (M2 macrophages), and CD3 (T cells). These markers were combined with fluorescence from calcein, which identifies viable cells. Mechanical disruption and digestion of the heart tissue as optimized in our protocol breaks cardiomyocytes apart, so the live cells are all non-myocytes, representing endothelial cells, smooth muscle cells, fibroblasts, and any hematopoietic cells that may have entered the heart. In Figure 1, it can be seen that the number of CD45^+^CD301^+^ M2 macrophages increased after I/R, as did the number that were CD45^+^CD301^+^collagen type I^+^ (and 38 ± 7% of the CD45^+^CD301^+^ were positive for collagen type I), consistent with our previous histological results that these cells are in the heart and are making collagen (13, 14). In addition, it can be seen that there were also T lymphocytes, identified by their CD45 and CD3 markers. Of note, in human heart tissue from cases of cardiomyopathy, we also found CD45^+^ cells in a perivascular location expressing either a myofibroblast marker (α-smooth muscle actin) or the T cell marker CD3, as well as CD301^+^ α-smooth muscle actin^+^ cells (Figure 2).

### Murine cytokines

**Figure 3:**
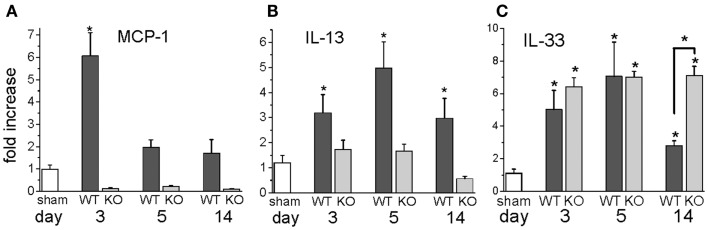
**Gene expression levels for MCP-1, IL-13, and IL-33 in wild-type (WT) or MCP-1 knockout (KO) after the indicated number of days of I/R, expressed as the fold increase over sham levels**. **(A)** the fold increase of MCP-1 in WT and KO animals over sham levels; **(B)** the fold increase of IL-13 in WT and KO animals over sham levels; **(C)** the fold increase of IL-33 in WT and KO over sham levels (*n* = 3 for WT and for KO shams; there was no difference between these groups, so the numbers were combined for the sham levels shown in the graph; *p < 0.05). The graph for IL-13 is reprinted with permission from (27).

In our mouse model, we were interested in the temporal gene expression of MCP-1 and IL-13. Our results showed high levels of MCP-1 mRNA early during the I/R response that waned thereafter (Figure 3). The Th2 product, IL-13, showed a later peak, but was present throughout the 2-week period of I/R. Neither of these responses took place in mice deficient in MCP-1 expression (genetic knockout, KO). IL-33, another mediator of interest because of its ability to induce IL-13, was increased with a similar pattern to that of IL-13. Unlike IL-13, IL-33 was induced in both the wild-type (WT) and the KO mice, and its expression was maintained at 14 days in the KO but not the WT mice.

We also measured specific cytokines that would characterize the T cells in the mouse heart as Th1 or Th2 by performing protein arrays on sham and I/R hearts after 3 days of treatment (Table 1). The Th2-related IL-13 protein was the most elevated protein in I/R over sham levels. The Th2 cytokine IL-4, by contrast, was not as increased, and neither was the major Th1 cytokine, IFN-γ.

**Table 1:** **Protein array data from hearts of animals subjected to 3 days of I/R**.

Cytokine	Fold change from sham^a^	Accession number
G-CSF	0.6 ± 0.2	NP_034101.1
GM-CSF	0.9 ± 0.1	NP_034099.2
IFN-γ	1.8 ± 0.4	NP_032363.1
IL-2	0.9 ± 0.1	NP_032392.1
IL-3	1.1 ± 0.2	NP_034686.2
IL-4	1.9 ± 0.6	NP_067258.1
IL-5	1.7 ± 0.3	NP_034688.1
IL-6	1.4 ± 0.3	NP_112445.1
IL-9	0.7 ± 0.1	NP_032399.1
IL-10	1.0 ± 0.2	NP_034678.1
IL-12p40p70	1.1 ± 0.1	NP_032378.1 (p40)
IL-12p70	1.3 ± 0.1	NP_001152896.1 (p35)
IL-13	3.7 ± 0.9	NP_032381.1
IL-17	2.4 ± 0.8	NP_034682.1
MCP-1	1.5 ± 0.2	NP_035463.1
MCP-5	0.8 ± 0.1	NP_035461.2
RANTES	1.1 ± 0.1	NP_038681.2
SCF	1.7 ± 0.2	NP_038626.1
sTNFR1	2.2 ± 0.5	NP_035739.2
TNF	2.0 ± 0.7	NP_038721.1
Thrombopoietin	3.1 ± 0.9	NP_033405.1
VEGF	1.9 ± 0.6	NP_001020421.2

^a^ Cardiac protein densities were obtained from three sham and three I/R animals normalized to the array positive controls. Densities from I/R animals were divided by the mean densities of the shams and averaged. Only proteins that gave a positive signal on the array are included.

### Human *in vitro* model

**Figure 4:**
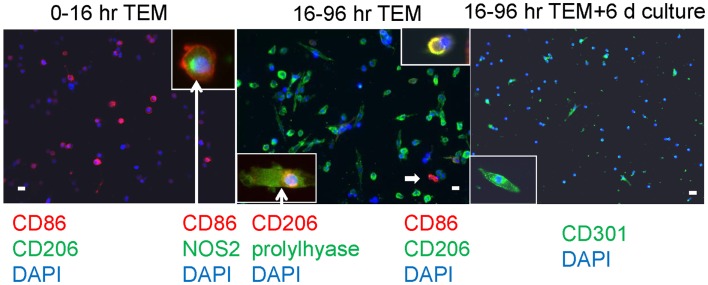
**Human PBMC were allowed to migrate for the indicated times through human cardiac microvascular endothelium in response to MCP-1 and thereafter adhered to poly d-lysine coated coverslips**. The cells were stained for the external markers CD86, CD206, and CD301, and after fixation and permeabilization, for the internal markers NOS2 and prolylhydroxylase (labeled prolylhyase in the figure). DAPI was used to stain cell nuclei blue. The insets are cells at either higher magnification (CD301) or cells with external staining and an internal marker (CD86 plus NOS2 and CD206 plus prolylhydroxylase) with white arrows extending from the label. The white arrow within the center panel indicates a CD86 positive cell in the low magnification field, and the inset at top right shows a cell that is double positive for CD86 and CD206. White bars represent 20 μm.

In our human *in vitro* TEM assay, we stained macrophages for M1 versus M2 markers after two different times of migration, early (migrating from 0 to 16 h) and late (cells migrating during the interval of 16–96 h), plus a group with an additional 6 days of culture post-migration. Macrophages developing from monocytes that had migrated early had a round morphology with markers of M1 macrophages, CD86 and NOS2 (nitric oxide synthase 2 or inducible NOS) (Figure 4). Cells bearing CD86 were 81 ± 2% of the total cells counted (out of 1038 counted from three donors). No cells bearing CD150, CD206, or CD301 were found at the early time interval. Many macrophages from monocytes that migrated later were elongated and had an M2 marker, CD206, as well as making prolylhydroxylase (the last enzyme in the pathway of collagen synthesis). Cells positive for CD206 were 64 ± 5%, for CD86 were 8 ± 2%, and double positive for CD206 and CD86 were 5 ± 1% of the total cells (1019), indicating the possibility of M1 to M2 conversion. No CD150 or CD301 positive cells were found in this time frame. With further culture (6 days), some of the elongated cells became positive for CD301, another marker for M2 macrophages (24). Cells positive for CD301 were 23 ± 5% of the total (471). No CD150^+^ cells were seen. Cells positive for CD206 were 61 ± 4, for CD86 were 3.4 ± 0.7, and double positive were 1.9 ± 0.5 out of a total of 924.

### Human cell identification

**Figure 5:**
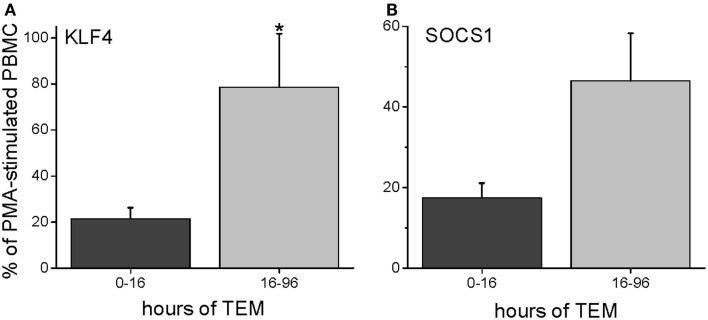
**Human PBMC transmigrated for 0–16 or 16–96 h were expanded with mitogens (PMA and calcium ionomycin) and harvested for RNA**. The gene expression levels were expressed as a percentage of mRNA levels in unmigrated mitogen-expanded total PBMCs (PMA = 100%), giving the percent levels of KLF4 **(A)** or SOCS1 **(B)** (*n* = 3 donors in duplicate).

**Figure 6:**
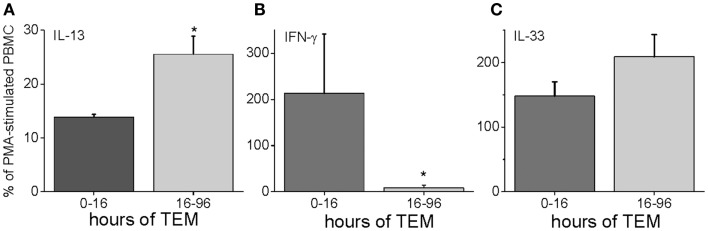
**Human PBMC transmigrated for 0–16 or 16–96 h were expanded with mitogens and harvested for RNA**. Their gene expression levels were expressed as a percentage of mRNA levels in unmigrated mitogen-expanded total PBMCs for IL-13 **(A)**, IFN-γ **(B)**, and IL-33 **(C)** (*n* = 3 donors in duplicate, *p < 0.05 for the comparison of the indicated group from the total PBMC level).

To characterize further the kinetics of macrophage M2 development, we performed quantitative PCR on *in vitro* human transmigrated cells from two different time frames (0–16 and 16–96 h). The cells were expanded with the mitogens phorbol myristate acetate (PMA) and calcium ionomycin. To relate gene expression to a negative control (either unmigrated cultured total leukocytes or freshly isolated uncultured mononuclear cells) would not have been productive because many genes are not expressed in these cells. Therefore, we calculated the gene expression as a percentage of the theoretical maximum expression from the same donor’s total unmigrated mononuclear leukocytes cultured with mitogens. The mitogen-induced signals from the total cells should not be biased toward one lineage because they contain all of the starting cell populations. It can be seen in Figure 5 that two transcription factors involved in M2 development, KLF4 and SOCS1 (25, 26), were present early but also increased with later transmigration. Flow cytometry indicated that CD3^+^ T cells had also transmigrated, and their distribution of CD4 and CD8 subsets after 4 days of migration was similar to that of the original T cells (fresh CD3^+^ MNLs were 73 ± 4% CD4^+^ and 24 ± 7% CD8^+^ while transmigrated CD3^+^ cells were 69 ± 3% CD4^+^ and 22 ± 6% CD8^+^, *n* = 3). An analysis of their gene expression showed increased levels of IL-13 mRNA with time [Figure 6 and as we have found in the *in vivo* model (27)] in conjunction with dramatically decreased levels of IFN-γ mRNA (Figure 6). These data indicated that the lymphokine kinetics were compatible with an early Th1 response (IFN-γ) followed by a Th2 response (IL-13) and downregulation of Th1 gene expression.

IL-13 can also be upregulated by IL-33, which is a member of the IL-1 family (28, 29). We measured the gene expression of IL-33 in our human model, and found that it was highly expressed both early and late during TEM (Figure 6).

## Discussion

Under homeostatic regulation, inflammation is followed by its resolution (1). In infections, removal of the responsible agent leads to the quieting of the immune response. In injury, cell death by various modes leads to active anti-inflammatory signaling. In our *in vivo* and *in vitro* models, in which cell death is avoided, it is clear that the same transition from proinflammatory to anti-inflammatory responses can take place, as well as a subsequent profibrotic environment. In both simplified *in vivo* and *in vitro* models, the initiating factor is MCP-1, considered a proinflammatory signal, but the end stage is fibrosis. In the *in vivo* model, the shift from inflammation to fibrosis may be mediated at least partially by IL-13, which is well known for this effect (30, 31). Indeed, the targets of IL-13, M2 macrophages, were present in the heart at that time (3 days). Some of these cells were making collagen, indicating that they had already responded to profibrotic mediators. The source of the IL-13 may be the CD3^+^ T cells that transmigrated in response to MCP-1, because IL-13 was lacking in the MCP-1 knockout. We also demonstrate that T cells were found in the human heart with cardiomyopathy; this indicates that our *in vivo* model may be relevant to human disease. Another stimulus for the upregulation of IL-13 in WT animals may be increased IL-33, expected to be made by infiltrating immune cells. However, IL-33 gene expression occurred even in the absence of MCP-1. IL-33 can be secreted from cells under mechanical strain (32), and is constitutively expressed in endothelial cells (33), so these may be sources of IL-33 that are independent of an inflammatory infiltrate. In the MCP-1 KO animals, IL-33 gene expression did not decline at the 14-day time point, indicating the possibility that MCP-1 could be responsible for the eventual downregulation of IL-33 mRNA.

As in the murine *in vivo* model, the presence of MCP-1 in the human *in vitro* model induced first a Th1 (IFN-γ) and M1 (CD86, NOS2) response that within a day shifted to a Th2 (IL-13) and M2 (CD206, KLF4, and SOCS1) plus fibrotic (prolylhydroxylase) response without the addition of any further mediators. Therefore, the proinflammatory response uncomplicated by known external stimuli for its resolution was down regulated and evolved into a fibrotic response. A resolution to this conundrum may be found in further investigation of the action of MCP-1.

The view of MCP-1 as being responsible only for inflammation has been challenged by findings in other models; effects were noted either directly on myeloid cells or indirectly via T cell production of cytokines. Human monocytes *in vitro* and in tumor environments can be induced by MCP-1 to undergo M2 polarization (34). T cells treated with MCP-1 elaborate IL-4 and IL-13 (Th2 cytokines) (35, 36) and MCP-1-deficient mice cannot mount Th2 responses (37). Some of the influence of MCP-1 on the promotion of Th2 responses may be mediated through MCP-1-induced protein-1 (MCPIP1), a down regulator of inflammatory responses that degrades cytokine mRNAs and deubiquitinates TNF receptor-associated factor family proteins (38, 39), thus allowing an unfettered Th2 response. MCP-1 may induce both inflammation and anti-inflammation, perhaps at different times or in different environments, and the cessation of its proinflammatory effects may operate through the induction of anti-inflammatory gene products.

In previous studies of myocardial infarction injury (with extensive cell death), the presence of two monocyte subsets, one inflammatory and the other reparative, was noted in mouse models (40) and in human patients (41). In myocardial infarction, the proinflammatory macrophages are thought to be required for terminal digestion of tissue and removal of necrotic debris, which would not be necessary in our models. Later after infarction, reparative macrophages (M2) dominate. Thus, there is the generation of a temporal pattern such that the influx of inflammatory macrophages (phenotypically and functionally identical to M1 macrophages) early after injury is followed by reparative macrophages (identified as M2) (11, 42). In our models, despite the absence of cardiac injury sufficient to cause cell death, there was a reprise of this temporal pattern, including a transient M1 response followed by a robust M2 response, despite there being nothing to “repair.” In fact, our previous studies demonstrated a transition to a state of unrestrained fibrosis resulting from dysregulation of immune function in M2 macrophages (14, 21). One cause may be that IL-13 can drive further production of MCP-1, which may set up a profibrotic feed forward loop (43). Thus the fibrotic response may represent dysregulation of a potentially protective immunological evolution (21).

Members of the adaptive immune system recognize foreign or altered self molecules via their multichain immunoreceptors, and innate immune cells recognize conserved pathogen-associated molecules through their pattern recognition receptors. In the absence of infection or cognate antigen presentation, however, there can be cytokine-induced cytokine production by both conventional and innate lymphoid cells (44). For example, the IL-1 family member IL-33 (an alarmin) causes antigen-independent IL-13 production (28, 29). IL-33 gene expression was present in both our models, although we do not know if this was an ancillary or primary stimulus for IL-13 production. The lack of IL-13 and presence of IL-33 in the MCP-1 knockout animals may be interpreted as the production of IL-33 in resident cells, which thereafter cannot induce the production of IL-13 from an absent inflammatory infiltrate. In either case (antigen recognition or cytokine-induced), we have demonstrated that MCP-1 alone can initiate an immune response that moves rapidly through a Th1/M1 inflammatory stage and into a Th2/M2 anti-inflammatory and profibrotic environment. Thereafter, the establishment of a chronically fibrotic state may depend on dysregulation of the immune response, as in diseases such as systemic sclerosis (45). Although MCP-1 and its receptor CCR2 historically have been targeted to reduce inflammation [reviewed in (46)], the possibility that it may initiate chronic fibrosis without chronic inflammation has not been considered. Our simplified models may allow an examination of this issue, as we propose that MCP-1 may initiate both an M1 and an M2 response leading to fibrosis, or promote M1 to M2 conversion.

**Figure 7:**
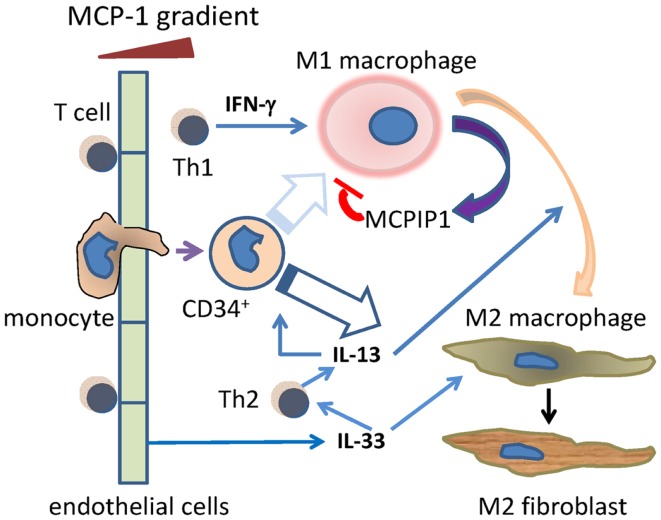
**Schema of the Th1/M1 and Th2/M2 responses after cells move through blood vessel endothelium**. Monocytes and T cells migrate through endothelium, initially in response to MCP-1, and including a primitive (CD34^+^) monocyte. The Th1 product IFN-γ drives the migrated monocytes toward the M1 phenotype, although this may be less likely for the CD34^+^ subset (faded arrow). MCP-1 itself induces migrated activated macrophages to secrete MCPIP1 (MCP-1-induced protein-1), which limits the activated M1 phenotype. During that initial Th1/M1 phase, Th2 activities such as the secretion of IL-13 begin, and become dominant at later time points. This may be aided by endothelial cell secretion of IL-33. IL-13 may promote some M1–M2 transition as well as direct monocyte to M2 maturation. The resulting M2 macrophages can become collagen-secreting myeloid fibroblasts.

The data from a murine model with daily episodes of brief ischemia not inducing infarction and an *in vitro* model of mononuclear cell TEM driven by MCP-1 both demonstrate the presence of a sequential inflammatory reaction occurring without evidence of tissue injury or infection and leading to interstitial fibrosis. The reaction is initiated by MCP-1-induced TEM of mononuclear cells (monocytes and T lymphocytes) and does not occur in the absence of MCP-1. Initially, the mononuclear cells are predominantly in an inflammatory phenotype (M1, Th1) and secrete cytokines and lymphokines appropriate to that phenotype (Figure 7).

As the reaction progresses, mononuclear cells of an “anti-inflammatory” phenotype begin to appear and subsequently dominate the cell population. This is followed by the appearance of a population of fibroblasts expressing collagen while still bearing the markers of their myeloid origin (CD45) and alternative polarization (CD206, CD301). The data suggest that the signaling cascade also contains intrinsic “brake mechanisms” that limit the intensity and duration of the acute inflammatory response. From the data presented, we suggest potential signaling molecules that may orchestrate this “braking” response as well as those initiating the transition from M1 to M2 phenotypes (Figure 7). We also suggest that our previous findings of CD34^+^CD45^+^ cells infiltrating the mouse heart during injuries may provide evidence for a relatively primitive monocyte precursor for either or both the M1 and M2 macrophages, although the finding of collagen in these cells suggests that they may be destined preferentially for the M2 phenotype (13, 16).

As described above, IL-33 is a powerful inducer of IL-13 and the Th2 phenotype without requiring an antigen response. Our data suggest that it also may be an important factor in the initiation of a Th2 response in an area of inflammation without tissue necrosis. In addition, MCPIP1 is secreted from activated M1 macrophages and has been observed to block M1 signaling (47), so this modulator may also aid in the transition from a Th1/M1 to a Th2/M2 response. Our previous and current data suggest that the chronic presence of M2 macrophages is associated with some of them undergoing maturation into secretory myofibroblasts. The mechanism and signaling associated with this maturation has not been well characterized although the phenomenon has been described *in vivo* and *in vitro* (13, 17, 48, 49). When the stimulus continues, as it does in our *in vivo* model, even the basic response may change its nature to that of a chronic pathology (fibrosis) very different from the initial inflammation. Having thus identified the sequential immune responses and the initial signals involved, we will investigate further pathways and additional responses involved in chronic fibrosis in future studies.

## Materials and Methods

### Animals and ischemia reperfusion protocol

Ten to twelve week old B6.129S4-Ccl2^tm1Rol^/J (MCP-1-KO) and C57BL/6J WT mice (both from Jackson Laboratory) were subjected to closed chest surgery as previously described (12). Briefly, 1 week after suture implantation, 15-min occlusions of the left anterior descending artery were performed for the number of indicated days, allowing a 24-h reperfusion period in between. Mice were euthanized 5 h after the last ischemic episode. All mice were fed standard mouse chow and water *ad libitum*. The investigation conforms to the *Guide for the Care and Use of Laboratory Animals* published by the US National Institutes of Health. All animals were treated in accordance with the guidelines of the Baylor College of Medicine Animal Care and Research Advisory Committee.

### Human heart tissue

Tissue pieces from human hearts with cardiomyopathy were obtained under an Institutional Review Board-approved protocol (50). The formalin-fixed tissue was paraffin embedded and sectioned.

### Mouse cell identification

After 5 days of I/R, mice were anesthetized with 2% isoflurane followed by cervical dislocation. Hearts were isolated and non-myocytes were isolated by enzymatic digestion using 0.1 mg/ml Liberase TH Research Grade (Roche Applied Science), as described previously (13, 18). For flow cytometric analysis, 1–3 × 10^5^ freshly isolated cells were incubated with 0.5 μg biotin-conjugated anti-CD45 antibody (BD Biosciences) followed by PE/Cy-5-conjugated streptavidin (BD Biosciences), together with either 0.5 μg anti-CD301 (Abcam Inc) + PE-conjugated anti-rat secondary antibody (Jackson ImmunoResearch), or PE-conjugated anti-CD3ϵ (Beckman Coulter). Fifty nanomolars calcein^AM^ (Invitrogen Molecular Probes) was added to measure cell viability (only live cells metabolize calcein to an intracellular, green-fluorescent permanent cell dye). A subset of cells were incubated with antibodies against CD45 and CD301 as described above, then fixed and permeabilized (kit from BD Pharmingen), and incubated over night with 1 μg anti-collagen type I antibody (Rockland), followed by FITC-conjugated anti-rabbit secondary antibody (Jackson ImmunoResearch). Fluorescence intensities were measured on a Beckman Coulter Epics XL.MCL using EXPO32 software.

### Human cell identification

Human heart sections were stained with H&E according to standard procedures (12). Before immunofluorescence staining, tissue sections were autoclaved in citrate buffer pH 6 for antigen retrieval and then permeabilized in 1% Triton X-100; after staining, sections were exposed to 0.3% Sudan Black in 70% alcohol to quench autofluorescence. Tissue sections were stained as described previously (17, 18) using anti-CD45 (BD Biosciences), -α-SMA (Sigma), -CD3ϵ (Beckman Coulter), and -CD301 (Dendritics).

Cells that transmigrated through an endothelial barrier were allowed to attach to poly d-lysine-coated coverslips (Neuvitro) at the bottom of the lower well. The cells were fixed and stained with anti-CD86 (BioLegend), -CD301 (Dendritics), or appropriate isotype controls. For the internal antigens, the cells were also permeabilized (BD Pharmingen) and stained with anti-prolylhydroxylase (Millipore), -CD206 (Epitomics), or -NOS2 (Abcam Inc). These were followed by affinity-purified F(ab′)_2_ IgG specific fluorochrome-conjugated secondary antibodies (Jackson ImmunoResearch) and the coverslips were mounted with the cell nucleus stain DAPI. Microscopy was performed on an Olympus AX70 using a QImaging Retiga 2000R camera. Colors were assigned and merged using ImageJ software (version 1.46r, NIH).

For cytometry, mononuclear leukocytes were stained fresh or after a 4-day transmigration with anti-CD3 plus either anti-CD4 or -CD8 (all from R&D Systems). Cells were analyzed on a Cell Lab Quanta SC flow cytometer (Beckman Coulter) using the Quanta Analysis software.

### Protein array

After 3 days of I/R, hearts were harvested and protein was isolated using Cell Lysis Buffer (RayBiotech) supplemented with Halt Protease and Phosphatase Inhibitor Cocktail (Thermo Scientific). Protein (250 μg) was loaded onto standard mouse cytokine antibody array membranes (cytokine array C1, RayBiotech). Membranes were processed according to the manufacturer’s instructions, images on film were scanned, and densitometry was assessed by ImageJ software.

### Transendothelial migration

As detailed earlier (17, 18), normal blood was obtained from volunteers under a protocol approved by the Institutional Review Board of Baylor College of Medicine. Anticoagulated blood was fractionated by Ficoll-Hypaque gradient centrifugation (Histopaque-1077; Sigma) to collect mononuclear cells (PBMC). For a TEM assay, HCMEC (Lonza, passages 4–9) were seeded on insert membranes with 8 μm pores. Human PBMC [25 × 10^4^ in RPMI 1640 with no serum but with ITS-X (Sigma) and antibiotic-antimycotic (Invitrogen)] were then added to each insert and the same medium including 650 ng/ml MCP-1 (R&D Systems) for chemoattraction was added to the well below. PBMC were allowed to migrate for 16 h, and then the inserts were moved into new medium-containing wells (without additional MCP-1) for another 80 h (96 h total migration time, but separate from the 0–16 h time frame for migration). At the end of each migration period, the migrated cells were expanded in medium plus 5% FBS (HyClone) plus PMA plus calcium ionomycin (both from Sigma) for 3 days and then harvested for RNA.

### RNA isolation and transcriptional expression

Cells were lysed and total RNA was isolated with TRIzol reagent (Life Technologies) and treated with RNAse-free DNaseI (Qiagen). Complimentary DNA was synthesized from 1 μg of RNA with a Verso cDNA synthesis kit (Thermo Scientific) using random hexamer and oligo-dT primers (3:1). Real-time PCR amplification reactions were performed with SsoAdvanced^TM^ SYBR Green Supermix (Bio-Rad) in triplicate using a CFX96 thermal cycler (Bio-Rad). Cycling conditions (annealing temperature/time) were optimized for each primer pair. Gene expression was measured by the ΔΔCT method and was normalized to HPRT or 18S ribosomal RNA levels. Primers were designed using Primer 3 ^1^, checked for homologies using BLAST ^2^ and for secondary structures using the mfold server ^3^. All primer pairs were verified to adhere to the MIQE guidelines using a temperature gradient and serial dilutions (51). Primer sequences (all in 5′–3′):

Human:
  KLF4: ACTCGCCTTGCTGATTGTCT and CCCCAAAGTCAACGAAGAGA
  SOCS1: CGAGCATCCGCGTGCACTTT and GCAGCTCGAAGAGGCAGTCGAA
  IL-13: CAATGGCAGCATGGTATGG and AGAATCCGCTCAGCATCC
  IFN-γ: TCCCATGGGTTGTGTGTTTA and GGGTCACCTGACACATTCAA
  IL-33: ACCCAAATGCTACCACTGGA and TCCACACATGCTAAGCACTCTT
  HPRT-1: GACCAGTCAACAGGGGACAT and CTTGCGACCTTGACCATCTT
Mouse:
  IL-13: TCCAATTGCAATGCCATCTA and TGAGTCCACAGCTGAGATGC
  MCP-1: TCCACAACCACCTCAAGCACTTC and GGCATCACAGTCCGAGTCACAC
  IL-33: GGCTGCTTGCTTTCCTTATG and CCGTTACGGATATGGTGGTC
  18sRNA: ACCGCAGCTAGGAATAATGGA and GCCTCAGTTCCGAAAACCA
  HPRT-1: GCCCCAAAATGGTTAAGGTT and TTGCGCTCATCTTAGGCTTT

## Statistical Analysis

All data are expressed as mean ± SE. A two-tailed, unpaired Student’s *t*-test or Mann–Whitney test was used to determine a significant difference between two groups. The Kruskal–Wallis ANOVA test with Dunn’s post-test was used for three or more groups. A *p*-value <0.05 was considered statistically significant.

## Conflict of Interest Statement

The authors declare that the research was conducted in the absence of any commercial or financial relationships that could be construed as a potential conflict of interest.

## Footnotes

^1^http://frodo.wi.mit.edu/primer3

^2^http://blast.ncbi.nlm.nih.gov/Blast.cgi

^3^http://mfold.rna.albany.edu/?q=mfold/DNA-Folding-Form
